# Is rumination only negative? Effects of belief in a just world on PTSD symptoms in firefighters: a moderated mediation model

**DOI:** 10.3389/fpsyg.2026.1817291

**Published:** 2026-05-20

**Authors:** Peng Wu, Donglin Hu, Siyu Tian, Si Chen, Yang Pan

**Affiliations:** 1 School of Sport Education, Tianjin University of Sport, Tianjin, China; 2 College of Sports Industry and Leisure, Nanjing Sport Institute, Nanjing, China; 3 School of Nursing and Rehabilitation, Shandong University, Jinan, Shandong, China; 4 Department of Physical Education, Tianjin Medical University, Tianjin, China

**Keywords:** belief in a just world, firefighters, post-traumatic stress disorder, resilience, rumination

## Abstract

**Objective:**

Belief in a just world (BJW) is correlated with an individual's psychological experience in the face of disaster. However, the association between BJW and post-traumatic stress disorder (PTSD) in firefighters remains unclear. Moreover, the extent to which the association between BJW and PTSD is mediated by resilience and moderated by rumination has yet to be determined. This study investigated potential strategies for mitigating PTSD symptoms in firefighters from the cognitive perspective of belief and examined the relationship among BJW, resilience, PTSD symptoms, and rumination.

**Methods:**

A moderated mediation model, with resilience as a mediating variable and rumination as a moderating variable, was tested based on data from 2,156 firefighters.

**Results:**

BJW was significantly negatively correlated with PTSD symptoms. Resilience partially mediated the relationship between BJW and PTSD symptoms, and rumination moderated the relationship between BJW and PTSD symptoms. Of note, the association between BJW and PTSD symptoms was not significant in the low-rumination group, and the negative effect of BJW on PTSD symptoms was stronger in the high-rumination group.

**Conclusions:**

BJW was linked to PTSD symptoms in firefighters through resilience, and the relationship between BJW and PTSD symptoms was influenced by rumination. Therefore, encouraging the development of higher levels of BJW and enhancing resilience may be effective strategies for alleviating PTSD symptoms in firefighters. Rumination did not exert uniformly negative effects in the present study.

## 1 Introduction

Firefighters are frequently exposed to a variety of traumatic events, such as physical injuries and life-threatening situations, during rescue and emergency operations, which directly or indirectly affect their psychological and physiological wellbeing (Katsavouni et al., 2016; Shin et al., 2023). Due to their high level of exposure to traumatic events, firefighters are at greater risk of developing PTSD than the general population and many other occupational groups (Berger et al., 2012) and are more likely to experience PTSD-related symptoms (Bastug et al., 2019; Lebeaut et al., 2021; Zegel et al., 2022). PTSD not only contributes to mental health problems such as depression in firefighters, but severe PTSD may also lead to self-harm and suicidal behavior (Boffa et al., 2018; Park et al., 2023). In addition, firefighters with PTSD exhibit longer reaction times to trauma-related stimuli and abnormal activation in trauma-related brain regions (Lee et al., 2022). These adverse psychological and physiological changes may negatively affect firefighters' personal lives and occupational performance (Bastug et al., 2019). Furthermore, previous research has indicated that not only firefighters who meet the clinical threshold for PTSD diagnosis but also those with subthreshold PTSD symptoms may experience adverse outcomes, including burnout (Chiang et al., 2021). Therefore, close attention should be paid to PTSD symptoms in firefighters regardless of whether they meet the formal clinical diagnostic threshold.

Cognitive therapy has become an important approach for the treatment of PTSD symptoms (Ehlers et al., 2014; LoSavio et al., 2017). As a factor influencing individual value judgments and behavioral responses, differences in just-world beliefs may shape individuals' psychological attitudes and behavioral orientations when confronting adverse events (Tian et al., 2022; Du and Zhu, 2007). Thus, belief in a just world (BJW) may represent an important cognitive factor relevant to PTSD intervention. Specifically, individuals who believe that the world is fair and that people generally get what they deserve are considered to possess stronger BJW (Lerner and Miller, 1978). Wu et al. (2013) pointed out that an important adaptive function of BJW is that it helps individuals focus on the future rather than becoming trapped in present difficulties. Previous studies have also demonstrated that BJW can buffer the negative psychological effects of adverse events (Otto et al., 2006; Tian, 2019). This may be because individuals with stronger BJW are more likely to rationalize adverse experiences and respond to life stressors in a more adaptive manner (Kong et al., 2021). Additionally, Ma et al. (2022) found that BJW significantly influences individuals' emotion regulation strategies. Firefighters with difficulties in emotion regulation are more likely to develop PTSD symptoms (Leonard and Vujanovic, 2022). Moreover, negative emotional states constitute a prominent component of PTSD symptomatology and may be a key target in PTSD treatment among firefighters (An et al., 2022). Indeed, BJW may protect emotional functioning during disasters by reducing negative affect and enhancing positive affect (Wang et al., 2021). However, the relationship between BJW and PTSD symptoms in firefighters has not been empirically established. Therefore, this study proposes.

**Hypothesis 1:** BJW is negatively associated with PTSD symptoms among firefighters.

Resilience is a vital trait in firefighters' ability to cope with major or traumatic events (Bernabé and Botia, 2016; Prell et al., 2020), as individuals with higher resilience are better able to adapt to changing conditions and recover more rapidly from emergencies caused by natural or human-made disasters (Rutter, 1985, 1987). Accordingly, resilience has been widely recognized as an important protective factor promoting firefighters' psychological health and occupational development (Heydari et al., 2022). Previous studies have shown that BJW is an effective predictor of resilience (Wu et al., 2011). According to the personal resource hypothesis of BJW, just-world beliefs function as a positive cognitive resource (Wu and Li, 2014). This psychological resource may facilitate the development of resilience (Chen et al., 2022). In turn, resilience promotes individuals' capacity to exert greater effort when coping with stress and adversity, thereby facilitating positive adaptation (Luthar et al., 2000). Prior research has also demonstrated that resilience is significantly associated with lower PTSD symptoms in firefighters (Jozani et al., 2023; Senger et al., 2023). Based on the above findings, this study proposes.

**Hypothesis 2:** Resilience mediates the relationship between BJW and PTSD symptoms.

Rumination has been shown to be closely associated with both BJW (DŽuka et al., 2021) and PTSD (Blackburn and Owens, 2016). Rumination refers to repetitive and passive thinking about distressing experiences or emotions that interferes with daily functioning (Wong et al., 2023). Individuals who develop more severe PTSD symptoms following traumatic events tend to exhibit higher levels of rumination (Zhou and Wu, 2016). Negative rumination can further exacerbate PTSD symptoms (Moulds et al., 2020). Therefore, rumination is considered an important target in PTSD intervention (Cox and Olatunji, 2017). Although the world contains many unjust and random events, making BJW partly a positive “illusion” (Hafer and Bègue, 2005). Lerner and Miller (1978) argued that such positive illusions are psychologically important for maintaining mental wellbeing. Previous studies have suggested that individuals with higher levels of rumination may continuously reinforce and validate their just-world belief system during repetitive cognitive processing, particularly when they hold more positive metacognitive beliefs (Moulds et al., 2010). This repeated confirmation may help individuals interpret traumatic experiences in a more constructive and meaningful manner, thereby alleviating PTSD symptoms (Wu et al., 2015). Given the associations among BJW, rumination, and PTSD, this study proposes.

**Hypothesis 3:** Rumination moderates the direct association between BJW and PTSD symptoms.

**Figure 1:**
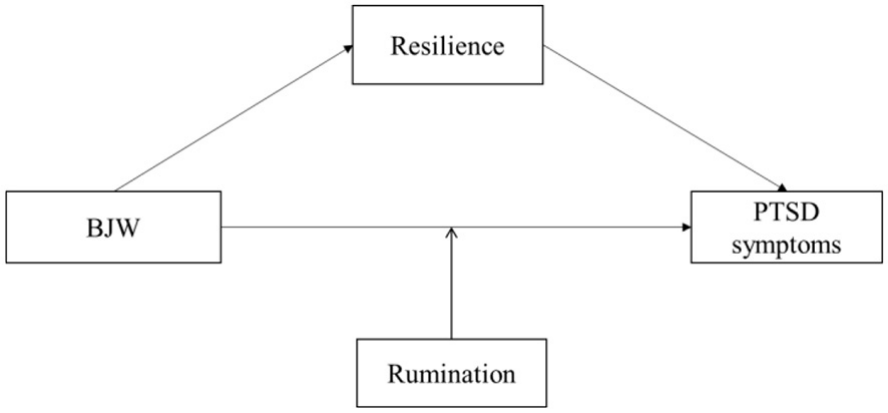
Research hypothesis model: the mediating role of resilience and the moderating role of rumination between BJW and PTSD symptoms.

In summary, this study aimed to examine the relationship between BJW and PTSD symptoms among firefighters and to explore the underlying mechanisms involved, with particular attention to the mediating role of resilience and the moderating role of rumination (see Figure 1). Specifically, this study tested the following hypotheses: (1) BJW is negatively associated with PTSD symptoms in firefighters; (2) resilience mediates the relationship between BJW and PTSD symptoms; and (3) rumination moderates the association between BJW and PTSD symptoms. The findings of this study are expected to provide theoretical support for the clinical intervention and treatment of PTSD symptoms in firefighters.

## 2 Materials and methods

### 2.1 Participants and procedure

In this study, a stratified cluster random sampling method was employed, and 22 fire brigades and 130 fire stations across China were selected as sampling units. Data were collected by contacting the commanders of each fire brigade in July 2022, through which 2,227 male firefighters were surveyed. The questionnaires required approximately 10 min to complete. Of these, 71 questionnaires were excluded from the analysis because of missing data. Ultimately, data from 2,156 male firefighters were included in the final analysis, yielding an effective response rate of 96.812%.

Specifically, the participants had a mean age of 26.94 ± 4.47 years, a mean length of service of 5.92 ± 4.37 years, and an average of 5.68 ± 7.01 emergency dispatches per week. Among them, 1,331 were unmarried, 811 were married, and 14 were divorced; 564 had children, whereas 1,592 did not. In addition, 904 participants resided in urban areas and 1,252 in rural areas. Regarding educational attainment, 459 had completed technical secondary school, 1,285 had junior college degrees, 392 had bachelor's degrees, and 20 had master's degrees or above. Furthermore, 390 participants had experienced occupational injury, whereas 1,766 had not. Detailed demographic characteristics are presented in Table 1.

**Table 1:** Basic characteristics of the research participants (*n* = 2,156).

Characteristics	Type	M ± SD/Number
Age (years)		26.94 ± 4.47
Years of service (years)		5.92 ± 4.37
number of emergency dispatches per week		5.68 ± 7.01
Marital status	Unmarried	1,331
	Married	811
	Divorced	14
Parental status	With children	564
	Without children	1,592
Residential background	Urban	904
	Rural	1,252
Educational level	Technical secondary school	459
	Junior college	1,285
	Bachelor's degree	392
	Master's degree or above	20
Injury history	Yes	390
	No	1,766

This study was reviewed and approved by the University Ethics Committee (No. 2022-R-99), as well as by the local fire department. Prior to date collection, participants were informed of the study's purpose, confidentiality, and anonymity, and informed consent was obtained. Participants completed the self-report questionnaires in fire stations under the guidance of trained researchers. Data were collected between July 15 and September 19, 2022.

### 2.2 Measures

#### 2.2.1 Just world belief scale

The Chinese version of the Just world belief scale, originally developed (Dalbert 1999) and revised by (Su et al. 2012), was used to measure participants' BJW and consists of 13 items. The scale employs a six-point Likert scale, ranging from 1 (“strongly disagree”) to 6 (“strongly agree”). Responses to all items were summed to calculate the total BJW score, with higher scores indicating stronger BJW. Previous research has demonstrated that the scale possesses satisfactory reliability and validity in Chinese populations (Su et al., 2012). The Cronbach's α coefficient for this study was 0.984.

#### 2.2.2 The 14-item resilience scale

Participants' resilience was assessed using the Chinese version of the 14-Item Resilience Scale (Qianyu and Tian, 2013). The scale contains 14 items and uses a seven-point Likert scale, ranging from 1 (“very inconsistent”) to 7 (“very consistent”). Higher scores indicate greater resilience. Previous studies have shown that the scale is a reliable instrument for assessing resilience in Chinese populations (Qianyu and Tian, 2013). The Cronbach's α coefficient for this survey scale is 0.982.

#### 2.2.3 Impact of event scale-revised

PTSD symptoms were assessed using the Impact of Event Scale-Revised (IES-R), which has been shown to be a valid measure of PTSD symptoms in Chinese populations (Guo, 2007). The IES-R consists of 22 items and employs a five-point Likert scale, ranging from 0 (“not at all”) to 4 (“extremely”). Higher scores indicate more severe PTSD symptoms. The Cronbach's α coefficient for this study was 0.983.

#### 2.2.4 Chinese version of Nolen-Hoeksema ruminative responses scale

Rumination was assessed using the Chinese version of the Nolen-Hoeksema Ruminative Responses Scale, originally developed by Nolen-Hoeksema et al. (2008) and revised by Han and Yang (2009). The scale contains 22 items and uses a four-point Likert scale, ranging from 1 (“never”) to 4 (“always”). Total scores were obtained by summing responses across all items, with higher scores indicating greater rumination. Previous studies have demonstrated that the scale has satisfactory reliability and validity in Chinese populations (Han and Yang, 2009). In this study, the Cronbach's α coefficient was 0.980.

### 2.3 Statistical analysis

Statistical analyses were conducted using SPSS version 24.0 (IBM, Armonk, NY, USA). Descriptive statistics were used to calculate the median and interquartile range of all variables. Spearman's correlation analysis was performed to examine associations among variables. The hypothesized model (Figure 1) was tested using the Hayes PROCESS macro (version 3.0) for regression analysis. In this model, BJW was specified as the independent variable, resilience as the mediating variable, PTSD symptoms as the dependent variable, and rumination as the moderating variable. Previous studies have shown that PTSD symptom levels in firefighters vary according to demographic characteristics (Sun et al., 2020). Therefore, demographic variables—including age, years of service, number of emergency dispatches per week, marital status, parental status, residential background, educational level, and injury history—were included as covariates in the analyses.

To further examine the moderating effect of rumination on the relationship between BJW and PTSD symptoms, a simple slope analysis was conducted. Specifically, this study examined the direct and indirect effects of BJW on PTSD symptoms through resilience and tested whether the direct effect of BJW on PTSD symptoms was moderated by rumination. All continuous variables were standardized before being entered into the moderated mediation model. The effects were estimated using 5,000 bootstrap samples, and 95% bias-corrected confidence intervals (CIs) were generated. An effect was considered statistically significant when the CI did not include zero. The significance level was set at *p* < 0.05.

## 3 Results

### 3.1 Common method bias testing

Harman's single-factor test was used to assess common method bias. Through exploratory factor analysis of the unrotated factor solution, five factors with eigenvalues greater than 1 were identified, and the first factor accounted for 37.899% of the total variance, which is below the 40% threshold criterion. This result suggests that common method bias was not a serious concern in the present study.

### 3.2 Descriptive statistics and correlation analysis

The median and interquartile range and correlation coefficients for BJW, resilience, PTSD symptoms and rumination are presented in Table 2. BJW was positively correlated with firefighters' resilience (*p* < 0.01) and negatively correlated with PTSD symptoms (*p* < 0.01) and rumination (*p* < 0.01). Firefighters' resilience was negatively correlated with PTSD symptoms (*p* < 0.01) and rumination (*p* < 0.01). PTSD symptoms were positively correlated with rumination (*p* < 0.01).

**Table 2:** Descriptive statistics and correlation matrix.

Variable	1	2	3	4
1. BJW	-			
2. Resilience	0.575^**^	-		
3. PTSD symptoms	−0.317^**^	−0.329^**^	-	
4. Rumination	−0.300^**^	−0.301^**^	0.537^**^	-
ME	64.000	76.000	22.000	33.000
IQR	22.000	34.000	10.000	22.000

^**^p < 0.01.

### 3.3 Mediation analysis

As shown in Table 3, the mediation analysis indicated that BJW was significantly associated with PTSD symptoms (β = −0.098, *p* < 0.001) and resilience (β = 0.552, *p* < 0.001). Resilience was also significantly associated with PTSD symptoms (β = −0.104, *p* < 0.001). Table 4 presents the indirect effect estimates based on 5,000 bootstrap samples. The results confirmed that resilience had a significant mediating effect on the relationship between BJW and PTSD symptoms (Effect = −0.057, 95% CI: −0.082 to −0.034).

**Table 3:** Regression results for mediation.

Path	β	*SE*	*t*	*p*
BJW—PTSD symptoms	−0.098	0.021	−4.598	< 0.001
BJW—resilience	0.552	0.018	30.653	< 0.001
Resilience—PTSD symptoms	−0.104	0.021	−4.829	< 0.001

**Table 4:** Mediating effects of resilience between BJW and PTSD symptoms.

Indirect effect	Effect	Bootstrap *SE*	Bootstrap 95% CI	
			Lower limit	Upper limit
Indirect effect	−0.057	0.012	−0.082	−0.034

### 3.4 Moderated mediation analysis

**Figure 2:**
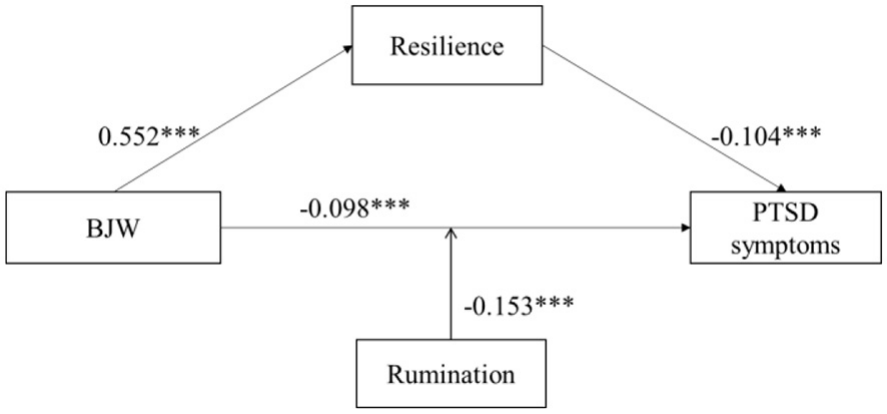
The path analysis results of the research hypothetical model. ***p < 0.001.

**Figure 3:**
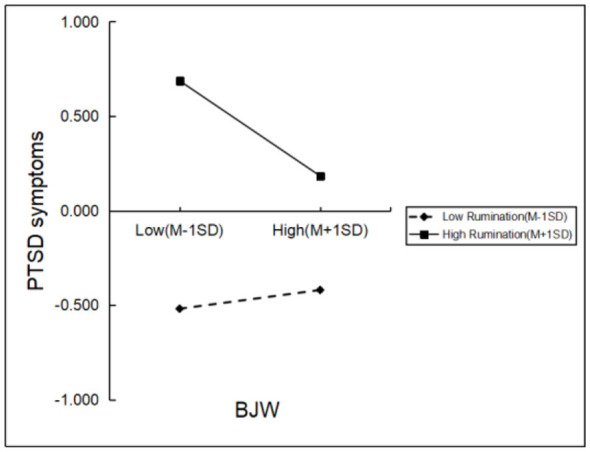
Moderating role of rumination on the relationship between BJW and PTSD symptoms.

As shown in Figure 2, the interaction between BJW and rumination in predicting PTSD symptoms was statistically significant (β = −0.153, *t* = −9.670, *p* < 0.001). To further clarify the moderating effect of rumination, a simple slope analysis was conducted. The results indicated that among firefighters with lower levels of rumination, the association between BJW and PTSD symptoms was not significant (*M* – 1*SD*; β = 0.049, *t* = 1.932, *p* > 0.05). In contrast, among firefighters with higher levels of rumination, the association between BJW and PTSD symptoms was significantly stronger (*M* + 1*SD*; β = −0.252, *t* = −9.177, *p* < 0.001). Detailed results are presented in Figure 3 and Table 5.

**Table 5:** Conditional direct effect at specified values of rumination.

Rumination	Direct effect	*SE*	*t*	*p*	95% CI	
					Lower limit	Upper limit
−1 *SD*	0.049	0.026	1.932	0.053	−0.001	0.099
*M*	−0.098	0.021	−4.598	< 0.001	−0.140	−0.056
+1 *SD*	−0.252	0.027	−9.177	< 0.001	−0.305	−0.198

## 4 Discussion

This study explored the relationship between BJW and PTSD symptoms, as well as the mediating role of resilience and the moderating role of rumination in this relationship. The results indicated that BJW was a significant negative predictor of PTSD symptoms among firefighters. Further analysis demonstrated that resilience partially mediated the relationship between BJW and PTSD symptoms. These findings support Hypotheses 1 and 2 and are consistent with previous studies (Otto et al., 2006; Tian, 2019; Tian et al., 2022). The findings suggest that BJW can predict PTSD symptoms both directly and indirectly through resilience. Individuals with higher BJW are more likely to rationalize adverse experiences and develop more positive attitudes toward traumatic events than those with lower BJW (Kong et al., 2021; Ulaş Karaahmetoglu and Durmus Iskender, 2024). Therefore, firefighters with higher levels of BJW may be less likely to develop PTSD symptoms. In addition, resilience serves as an important bridge linking BJW and PTSD symptoms. BJW increase individuals' sense of control and is positively associated with functional coping strategies (Karmakar, 2022; Yu et al., 2018). These resources help individuals achieve positive adaptation when facing adversity and may enhance firefighters' resilience (Chen et al., 2022), thereby enabling them to better cope with traumatic events and reducing the likelihood of PTSD symptoms (Jozani et al., 2023; Senger et al., 2023).

The present study also found that rumination moderate the direct relationship between BJW and PTSD symptoms. Specifically, BJW had no significant effect on PTSD symptoms among individuals with low levels of rumination. By contract, among individuals with high levels of rumination, BJW demonstrated a significantly stronger protective effect against PTSD symptoms. This finding indicates that individual differences exist in the cognitive mechanisms underlying PTSD symptoms and further highlights rumination as an important factor associated with PTSD symptoms, consistent with previous research (Blackburn and Owens, 2016). Although rumination is often regarded as maladaptive, as a repetitive cognitive process it may not be uniformly detrimental (Cann et al., 2011). According to Cann et al. (2010), reflective re-evaluation of beliefs is relevant to psychological adjustment following traumatic experiences. BJW may encourage firefighters to interpret traumatic experiences more positively (Lerner and Miller, 1978). Firefighters with high rumination may repeatedly reinforce positive metacognitive beliefs during rumination, which may buffer the psychological distress caused by traumatic experiences and reduce vulnerability to PTSD symptoms (Furnham, 2003). Accordingly, high levels of rumination may interact with BJW to strengthen its protective effect against PTSD symptoms.

This study provides meaningful theoretical and empirical contributions to the literature on PTSD symptoms among Chinese firefighters and offers practical implications for future research in this area. Given that firefighters represent one of the occupational groups at highest risk for PTSD and that PTSD symptoms may have severe long-term consequences for physical health, mental health, occupational performance, and quality of life, prior research has insufficiently examined the cognitive mechanisms underlying PTSD in this population. By systematically investigating the interrelationships among BJW, resilience, rumination, and PTSD symptoms, this study expands the current understanding of PTSD-related cognitive mechanisms in firefighters. Importantly, the findings suggest a novel perspective: rumination, traditionally conceptualized as a maladaptive cognitive style, may under certain conditions interact with belief-based cognitive resources such as BJW to enhance resistance to PTSD symptoms. This finding should be interpreted cautiously, as the adaptive function of rumination may depend on its cognitive content and contextual characteristics.

Several limitations should be acknowledged. First, this study employed a cross-sectional design, with data collected in 2022, which limits the ability to establish causal relationships and assess the long-term stability of the findings. Additionally, the temporal context of data collection may influence the applicability of the findings to current circumstances. Second, the sample consisted exclusively of Chinese male firefighters. Therefore, cultural background and gender may limit the generalizability of the findings to Western populations and female firefighters. Third, this study did not collect data on the types or frequencies of trauma experienced by participants, nor were trauma-related exclusion criteria established during sampling. Consequently, some participants may have had pre-existing mental health conditions, including PTSD, which may have influenced the finding. Future studies should consider adopting longitudinal or experimental designs to further examine the causal relationships and long-term validity of these findings. In addition, expanding the sample to include international and female firefighter populations would enhance the generalizability of the results and facilitate cross-cultural and gender-based comparisons. Future research should also incorporate detailed assessments of trauma type and frequency to further clarify the complex relationships among trauma exposure, BJW, resilience, rumination, and PTSD symptoms.

## 5 Conclusion

The results of this study indicate that BJW is negatively associated with PTSD symptoms among firefighters, and this relationship is partially mediated by resilience. Moreover, rumination moderates the relationship between BJW and PTSD symptoms, such that higher levels of rumination strengthen the association between BJW and PTSD symptoms. By examining the relationships among BJW, resilience, rumination, and PTSD symptoms, this study provides new theoretical insights and empirical evidence for the prevention and treatment of PTSD symptoms among firefighters. Specifically, enhancing BJW and resilience, while paying closer attention to rumination, may represent promising strategies for alleviating PTSD symptoms in this population.

## Data Availability

The original contributions presented in the study are included in the article/Supplementary material, further inquiries can be directed to the corresponding author.
