# Supplementary material for: Is rumination only negative? Effects of belief in a just world on PTSD symptoms in firefighters: a moderated mediation model
Source: Front Psychol. 2026 May 20;17:1817291. doi: 10.3389/fpsyg.2026.1817291 (PMC13230100; doi:10.3389/fpsyg.2026.1817291)
Supplement: Supplementary file 1 [file Table_1.docx]

| Impact of Event Scale-Revised | | |  |
| --- | --- | --- | --- |
| Item | factor loading | |  |
| Item 1 | 0.821 | |  |
| Item 2 | 0.759 | |  |
| Item 3 | 0.865 | |  |
| Item 4 | 0.821 | |  |
| Item 5 | 0.866 | |  |
| Item 6 | 0.894 | |  |
| Item 7 | 0.873 | |  |
| Item 8 | 0.878 | |  |
| Item 9 | 0.911 | |  |
| Item 10 | 0.885 | |  |
| Item 11 | 0.897 | |  |
| Item 12 | 0.911 | |  |
| Item 13 | 0.861 | |  |
| Item 14 | 0.899 | |  |
| Item 15 | 0.838 | |  |
| Item 16 | 0.919 | |  |
| Item 17 | 0.899 | |  |
| Item 18 | 0.878 | |  |
| Item 19 | 0.853 | |  |
| Item 20 | 0.836 | |  |
| Item 21 | 0.86 | |  |
| Item 22 | 0.875 | |  |
| The 14-Item Resilience Scale | | | |
| Item | | factor loading | |
| Item 1 | | 0.884 | |
| Item 2 | | 0.853 | |
| Item 3 | | 0.914 | |
| Item 4 | | 0.734 | |
| Item 5 | | 0.870 | |
| Item 6 | | 0.939 | |
| Item 7 | | 0.922 | |
| Item 8 | | 0.926 | |
| Item 9 | | 0.924 | |
| Item 10 | | 0.936 | |
| Item 11 | | 0.945 | |
| Item 12 | | 0.913 | |
| Item 13 | | 0.930 | |
| Item 14 | | 0.941 | |

| Chinese version of Nolen—Hoeksema Ruminative Responses Scale | |
| --- | --- |
| Item | factor loading |
| Item 1 | 0.818 |
| Item 2 | 0.805 |
| Item 3 | 0.846 |
| Item 4 | 0.848 |
| Item 5 | 0.849 |
| Item 6 | 0.887 |
| Item 7 | 0.858 |
| Item 8 | 0.892 |
| Item 9 | 0.896 |
| Item 10 | 0.899 |
| Item 11 | 0.852 |
| Item 12 | 0.755 |
| Item 13 | 0.709 |
| Item 14 | 0.854 |
| Item 15 | 0.858 |
| Item 16 | 0.763 |
| Item 17 | 0.891 |
| Item 18 | 0.765 |
| Item 19 | 0.869 |
| Item 20 | 0.847 |
| Item 21 | 0.866 |
| Item 22 | 0.876 |

| Just World Belief Scale | |
| --- | --- |
| Item | factor loading |
| Item 1 | 0.813 |
| Item 2 | 0.865 |
| Item 3 | 0.882 |
| Item 4 | 0.863 |
| Item 5 | 0.856 |
| Item 6 | 0.897 |
| Item 7 | 0.775 |
| Item 8 | 0.908 |
| Item 9 | 0.906 |
| Item 10 | 0.937 |
| Item 11 | 0.900 |
| Item 12 | 0.936 |
| Item 13 | 0.917 |
